# Supplementary material for: Drainage of inflammatory macromolecules from the brain to periphery targets the liver for macrophage infiltration
Source: eLife. 2020 Jul 31;9:e58191. doi: 10.7554/eLife.58191 (PMC7434444; doi:10.7554/eLife.58191)
Supplement: Transparent reporting form [file elife-58191-transrepform.pdf]

1st Floor
24 Hills Road
Cambridge CB2 1JP, UK

P 01223 855340
W elifesciences.org
T @elife

eLife's transparent reporting form

We encourage authors to provide detailed information *within their submission* to facilitate the interpretation and replication of experiments. Authors can upload supporting documentation to indicate the use of appropriate reporting guidelines for health-related research (see EQUATOR Network), life science research (see the BioSharing Information Resource), or the ARRIVE guidelines for reporting work involving animal research. Where applicable, authors should refer to any relevant reporting standards documents in this form.

If you have any questions, please consult our Journal Policies and/or contact us: editorial@elifesciences.org.

Sample-size estimation

- You should state whether an appropriate sample size was computed when the study was being designed
- You should state the statistical method of sample size computation and any required assumptions
- If no explicit power analysis was used, you should describe how you decided what sample (replicate) size (number) to use

Please outline where this information can be found within the submission (e.g., sections or figure legends), or explain why this information doesn't apply to your submission:

We determined an appropriate sample size based on the "resource equation" as described in "How to calculate sample size in animal studies?" https://www.ncbi.nlm.nih.gov/pmc/articles/PMC3826013/. This is an appropriate method as there is no assumption on the effect size or previous findings available for our study on a new biological process we have described between the brain and the liver leading to macrophage infiltration. Based on this calculation, we targeted a sample size of a minimum of 5-6 per group. Sample sizes are presented in the data throughout the Results section, and most studies range 10-20 per group amounting to a total of around 40-100 animals per experimental study.

Replicates

- You should report how often each experiment was performed
- You should include a definition of biological versus technical replication
- The data obtained should be provided and sufficient information should be provided to indicate the number of independent biological and/or technical replicates
- If you encountered any outliers, you should describe how these were handled
- Criteria for exclusion/inclusion of data should be clearly stated
- High-throughput sequence data should be uploaded before submission, with a private link for reviewers provided (these are available from both GEO and ArrayExpress)

Please outline where this information can be found within the submission (e.g., sections or figure legends), or explain why this information doesn't apply to your submission:

Experiments were repeated twice or more independently. Since this study involves individual animal brain injection, each experimental animal effectively also serves as an independent repeat of an experiment, so if accounting for this, experiments with a minimum of 10 animals for each group would represent ≥ 10 biological replicates. All results included in this study shown in main and supplementary figures represent biological replicates (each data point in graphs represent an individual animal or an individual cell analyzed). This pertains to fluorescent and brightfield imaging data as well as the qPCR results. For qPCR analysis, three technical replicates were used to derive each qPCR data point for an individual animal to control for pipetting and PCR errors; qPCR graphs shown in the Results are showing calculations for the biological replicates.

Statistical reporting

- Statistical analysis methods should be described and justified
- Raw data should be presented in figures whenever informative to do so (typically when N per group is less than 10)
- For each experiment, you should identify the statistical tests used, exact values of N, definitions of center, methods of multiple test correction, and dispersion and precision measures (e.g., mean, median, SD, SEM, confidence intervals; and, for the major substantive results, a measure of effect size (e.g., Pearson's r, Cohen's d)
- Report exact p-values wherever possible alongside the summary statistics and 95% confidence intervals. These should be reported for all key questions and not only when the p-value is less than 0.05.

Please outline where this information can be found within the submission (e.g., sections or figure legends), or explain why this information doesn't apply to your submission:

Statistical analysis methods are described throughout the Results section, and further summarized in the "Statistical Analysis" part of the Materials and Methods section.

(For large datasets, or papers with a very large number of statistical tests, you may upload a single table file with tests, Ns, etc., with reference to sections in the manuscript.)

Group allocation

- Indicate how samples were allocated into experimental groups (in the case of clinical studies, please specify allocation to treatment method); if randomization was used, please also state if restricted randomization was applied
- Indicate if masking was used during group allocation, data collection and/or data analysis

Please outline where this information can be found within the submission (e.g., sections or figure legends), or explain why this information doesn't apply to your submission:

Groups were allocated based on treatment groups, such as: uninjected, control vehicle injected, experimental molecule injected. In addition to treatment, some experiments included further categories based on genotype or molecular perturbation, such as: comparison between morpholino-injected and uninjected, and comparison between CRISPR cas9/gRNA injected versus uninjected animals. For data collection and analysis, the individual fish were either assigned numbers for analysis so the categories were blinded (not known to the experimenter), or that the groups were analyzed by intercalating control with experimental individuals to ensure equivalent assessment. Each graph in our paper represents an experiment that entails a number of groups based on treatment and genetic/molecular background. For each experiment, we used animals derived from the same clutches which were analyzed together on the same day in the same setting using the same tools and methods to ensure unbiased and equivalent data collection and processing among all animals in all groups for a reliable and accurate comparison. Animals were randomly chosen for each group allocation to eliminate any bias and accurately represent the population.

Additional data files ("source data")

- We encourage you to upload relevant additional data files, such as numerical data that are represented as a graph in a figure, or as a summary table

- Where provided, these should be in the most useful format, and they can be uploaded as “Source data” files linked to a main figure or table
- Include model definition files including the full list of parameters used
- Include code used for data analysis (e.g., R, MatLab)
- Avoid stating that data files are “available upon request”

Please indicate the figures or tables for which source data files have been provided:

All numerical data have been individually plotted in the graphs and figures of the Results section. All data are presented in the manuscript.
